# Are partnerships in nonprofit organizations being governed for sustainability? A partnering life cycle assessment

**DOI:** 10.1371/journal.pone.0249228

**Published:** 2021-03-29

**Authors:** Hazem S. Kassem, Salim Bagadeem, Bader Alhafi Alotaibi, Mohammed Aljuaid

**Affiliations:** 1 Department of Agricultural Extension and Rural Society, King Saud University, Riyadh, Saudi Arabia; 2 Department of Agricultural Extension and Rural Society, Mansoura University, Mansoura, Egypt; 3 Faculty of Business Administration, Arab Open University, Riyadh, Saudi Arabia; 4 Department of Health Administration, King Saud University, Riyadh, Saudi Arabia; Shenzhen University, CHINA

## Abstract

Goal 17 of the sustainable development goals (SDGs) attracted attention to the importance of partnerships between governments, the private sector, and nonprofit organizations (NPOs) for sustainable development. This paper aims to analyze the processes of establishing and operating the partnerships between NPOs and other actors in terms of governance. The best practices for partnership governance were examined according to the partnering life cycle framework. A simple random sample of 184 NPOs in six regions of Saudi Arabia was selected for data collection. These organizations were analyzed according to their governance practices in 937 partnerships established during 2016–2018. The findings showed that the organizations had strongly implemented the phases of building and scoping and managing and maintaining, while their governance practices regarding phases of reviewing and revising and sustaining outcomes ranged between moderate and low levels. The results also revealed significant differences between the overall implementation of the partnering life cycle practices and the NPO’s year of establishment. It was concluded that analyzing the current situation of implementing the best practices of partnership governance is useful to explore the efficiency and effectiveness of partnerships between NPOs and other actors, as well as the existing policy gaps, so as to create and implement sustainable-oriented partnerships.

## 1. Introduction

In 2015, the General Assembly of the United Nations (UN) formally provided a framework for "peace and prosperity for people and the planet, now and into the future", as formulated in the Global Sustainable Development Agenda [1, 2]. As part of this agenda, all United Nations member states, after a collaborative process involving multiple stakeholders, agreed upon the Sustainable Development Goals (SDGs) [3, 4]. The 17 SDGs are an urgent call to action by all countries to promote sustainable development in social, economic, and environmental aspects [5, 6]. SDG 17, to "strengthen the means of implementation and revitalize the global partnership for sustainable development", can be considered as a meta-goal for implementing the other SDGs [6, 7]. Global partnerships facilitate the necessary institutional and organizational structures to foster the transformational changes required to achieve multiple SDGs [4, 8]. These transformations require multi-stakeholder partnerships that mobilize and share financial resources, knowledge, technology, and expertise to address various complex problems [2, 7]. Therefore, collaboration between governments, the private sector, and nonprofit organizations (NPOs)—whether in intra-sectoral or inter-sectoral levels—is crucial to generate co-benefits [9, 10].

Globally, NPOs are a growing feature of societies and have become a key player as service providers of sustainable development concerns [2, 11]. These organizations operate independently and are not aimed at making a profit for shareholders, the board of directors, or an individual [12]. They have been called different names in different countries, including non-governmental organizations, civil society organizations, social economy organizations, third sector organizations, and voluntary organizations [13]. In general, they are classified into two broad categories: (i) social enterprises and (ii) charities and community groups [14]. According to Guo and Peng [15], the governance structures of these two types of institutions are their main distinguishing feature. Social enterprises are governed by persons or shareholders employed to work for the companies, while charities and community groups are governed by a community of volunteers who collaborate to achieve specific objectives [16]. As noted by Matic and AlFaisal [17], the legal structures in different countries affect the growth of these organizations and how they operate their activities to address the various social issues in society. Presently, NPOs act as catalysts for social change and are playing an increasingly important role in improving the standards of living of the population, including long-term security, equity, human development, and solving the major problems affecting people [18–22]. Furthermore, NPOs have played a significant role in helping public and private entities integrate sustainable development concerns into their decision-making processes [23, 24]. To facilitate social change processes that promote sustainability, NPOs engage with other actors in inclusive partnerships [2, 25–28].

Partnership is a powerful tool for collaboration between various parties, both public and private [29, 30]. A partnership reflects a dynamic relationship among diverse actors, based on a joint interest that stimulates their commitment to a complementarity of resources and value creation [10, 31]. A partnership encompasses mutual influence, with a careful balance between autonomy and synergy, incorporating equal participation in decision-making, mutual accountability and transparency, and mutual respect [32]. However, due to the enormous range of definitions and typologies that partnerships assume regarding the depth of their engagement [4], it is a common assumption that a partnership is based on sharing rather than transferring costs and risks [33, 34]. The type of sharing can range from a simple exchange of resources and competencies to a far more complex sharing of program development and delivery, decision-making, and governance [35, 36]. Diverse motives have been identified by the literature for partnerships between NPOs and other actors, including access to financial resources; integration for addressing complex societal problems; the effective and efficient delivery of programs; strengthening NPOs’ reputations and political influence, opportunities to reach a wider audience; learning and innovation and inter-agency co-ordination; pooling resources; increasing communication among groups and breaking down stereotypes; building networks and friendships; creating business value and environmental benefits; leveraging skills and perspectives not available in the organization; building respect and credibility; providing independent validation; and helping achieve a long-term vision [26, 37–40].

Partnerships between NPOs and other actors can operate at two levels: intra-sector (nonprofit–nonprofit) and inter-sector (business–nonprofit, government–nonprofit, and tri-sector partnerships) [26, 41]. Byiers et al. [6] suggested a framework to determine the characteristics and assess corporate–NPO partnerships according to four dimensions: (i) relationship with core business activities, (ii) degree of partners’ engagement, (iii) the partnership’s activities, and (iv) governance structures. Partnerships can be categorized according to whether their associated activities are related to the partners’ core business activities (strategic partnerships) or not (philanthropic partnerships) [42]. In terms of the degree of the partner’s engagement, strategic partnerships require a different degree of partner engagement in the (i) frequency of their interactions, (ii) intensity of their interactions, and (iii) types of resources shared compared to philanthropic partnerships [43]. Furthermore, the activities involved in philanthropic partnerships can be characterized by their simplicity, such as their marketing activities/campaigns, advocacy, donations and sponsorships, employee fundraising, and product licensing. On the other hand, strategic partnerships involve a set of more complex activities performed in regular operations, such as production, marketing, or new business development, which are described by the concept of value co-creation [40]. For governance structures, this dimension focuses on how the partnerships are set and managed and identifies the way that power is distributed within the partnership [44]. In this paper, we focus on partnerships as a specific governance arrangement between NPOs and other actors.

To develop successful collaborations between NPOs and other actors, building an effective collaborative governance structure for the partnerships is essential [45]. According to Gray and Stites [46], a governing arrangement refers to one or more institutions directly engaged in a collective decision-making process that is formal, deliberative, and consensus-oriented that aims to manage specific programs, assets, or services to achieve a common goal. A well-designed governance structure consequently has important implications for the partnership’s internal processes, decision-making processes, and outcomes [47]. In a similar sense, Ansell and Gash [48] argued that the implementation of collaborative governance is a means to emphasize that partnership as a two-way process. In other words, governance agreement reflects the commitment of that partnership to the partnership’s principles (shared vision, goals and projects, dialogue and mutual learning, community participation, accountability, integrity and transparency, no discrimination, and quality orientation), the commitment to consider the obligations of partners to their roles and responsibilities and make the relationship transparent, and the commitment to mutual capacity development [49–51]. Accordingly, analyzing the partnering life cycles of partnerships is crucial to determine the problems encountered in meeting the expectations of such partnerships [52]. These problems include (i) inconsistencies in performing operations and weak co-ordination, (ii) inefficiency in service delivery, and (iii) weaknesses in accountability due to blurred lines of responsibility [53].

In Saudi Arabia, the nonprofit sector comprises organizations and entities that provide services in nine main fields: culture and entertainment, education and research, health, social services, environment, development and housing, advocacy, charity, and religious education [54]. Over the past several years, the performance of NPOs has been characterized as ineffective and governed by traditional management practices. This weakness is represented in the following indicators: sharing in less than 1% of the GDP, a lower rate of NPO outreach (1:43,000), a decline in volunteers involved in NPO activities, a limited scale of professionalism and effectiveness, focusing on "charity" instead of social development, and a lower rate of employment contributions [55]. Furthermore, the sector has been faced with different challenges, including a lack of funding; networking with the private sector; aligning NPOs’ missions, methods, and resources; earning the public’s trust and creating awareness of the significance of nonprofits; balancing individual interests with the common good, and institutionalizing the internal functions and structures of NPOs [56]. In 2016, the Saudi government announced the vision of Saudi Arabia 2030. This vision pays more attention to strengthening the nonprofit sector’s social impact and enabling it to transform towards institutionalization and sustainability-oriented programs [57]. This vision aims to improve the contributions of the nonprofit sector to the GDP from less than 1% to 5% by the year 2030 [58]. The objectives of the vision are to overcome the legal constraints for establishing NPOs, promote cooperation between the institutions of the sector and government agencies, develop partnerships with other actors, implement standards of good governance, and change the culture of volunteerism among members of Saudi society [59]. In 2018, the number of NPOs rose by 131% from 2011 (1125) to 2018 (2598), as did their revenues (7.8%), expenses (8.3%), and assets (10.2%) [60, 61]. Furthermore, during the period of 2016–2019, there were increasingly significant partnerships established between NPOs and other actors to achieve the vision’s objectives [62].

Despite the growing interest in analyzing the various aspects of partnerships in the literature, limited evidence has been observed regarding the interactions of formal and informal governance processes and structures throughout the lifetime of a partnership, specifically in the context of Saudi Arabia. Accordingly, there is a need for more comprehensive empirical research to understand the governance practices and mechanisms that facilitate successful partnerships. The objectives of this study were to identify current NPOs’ implementation levels of governance practices during the life cycle of their partnerships and determine the differences in implementing these practices based on the NPOs’ characteristics.

## 2. Literature review

### 2.1. Governance for significant partnerships

Building successful partnerships between organizations is one of the most essential good corporate governance practices [63]. Good governance for sustainable-oriented partnerships is characterized by efficiency, effectiveness, participation, and transparency [64]. From a collaborative perspective, these characteristics can be achieved if partners apply corporate governance principles over the life cycle of the partnerships [65–67]. These principles include focusing on the organization’s mission and community needs; having clear responsibilities and arrangements for accountability; facilitating good conduct and behavior, informed and transparent decision-making, and managing risk before entering into a partnership; developing the skills and capacities of all representatives; and engaging stakeholders [68–70]. Applying the principles of governance is always the responsibility of the governing board [71]. Boards set the policies and strategies, make decisions, monitor organizational performance, and ensure organizational accountability to stakeholders [72, 73]. Consequently, the skills of the board members have a significant influence on organizational governance [74]. In this context, Renz [75] noted that NPO success depends on well-managed boards.

The governance of cross-sector partnerships has received growing academic interest due to the differences between partners in their configurations and objectives [76]. These partnerships face two interrelated challenges that may affect their effectiveness: (i) transforming relationships in the context of power asymmetries and (ii) stimulating long-term transformative relationship through short-term programs [77]. Sustainable-oriented partnerships are established with the principle of "power balance", where each partner has the opportunity to participate in achieving sustainable change [78, 79]. Bitzer and Glasbergen [80] argued, however, that partners tend to differ in terms of their control over resources when their interests are diverse. Power dynamics can have a range of unsatisfactory consequences, as low-power actors may be ignored, overruled, co-opted, or excluded by dominant actors [81]. However, there is no understanding as of yet about the practices, mechanisms, and strategies through which power asymmetries are addressed [82]. Therefore, good governance practices are required to define the substantive scope and conditions at the beginning of the partnership [83]. In terms of the timeframes of the partnerships, cross-sector collaborations are designed as short-term projects but aim to transform relationships in the long term [84]. As Grabher [85] noted, the limited timeframes of partnerships lead to interrupted and discontinued relationships. On the other hand, long-term relationships are a positive prerequisite for sustaining value creation [86, 87]. To conclude, both the power asymmetries and timeframes of partnerships should be well understood as "tensions" that appear through formal and informal structures and processes and may change over time [77]. In this regard, Hayes, Cornforth [88] indicated that these tensions are connected to the partnership’s effectiveness and directly affect those who implement governance activities. Hereafter, tensions must be addressed to ensure the sustainability and goal-achievement of the collaboration [89].

### 2.2. The partnering life cycle framework

**Fig 1:**
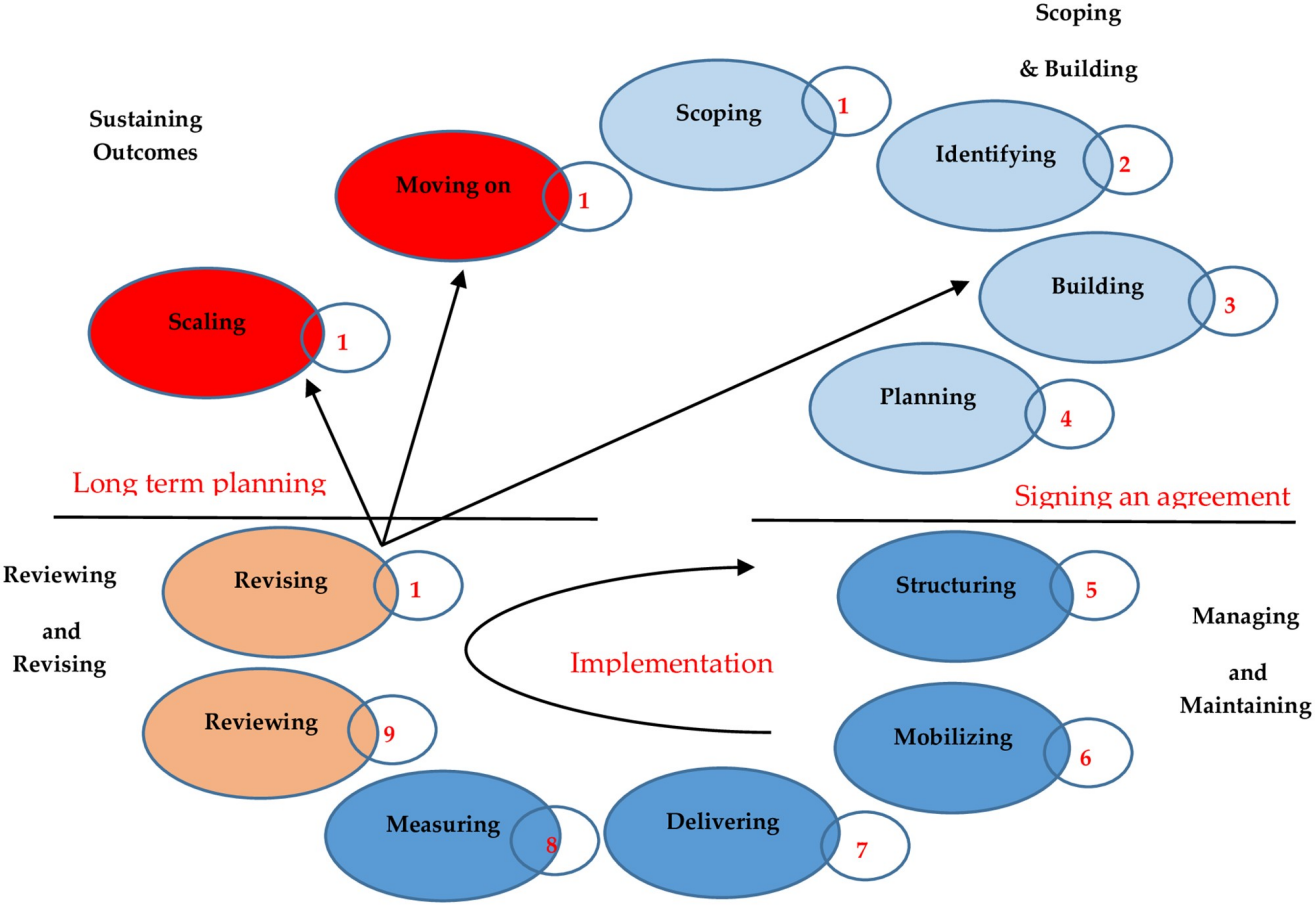
The partnering life cycle framework. Source: Tennyson (93).

To understand how tensions are addressed through governance, informal and formal governing mechanisms and their interactions throughout the partnership’s lifetime should be identified [88]. Formal governance outlines structures that define actors’ roles and obligations to each other, written in a legal form [90]. Formal processes are implemented through positions of authority (e.g., negotiations, steering, or controlling) [91]. By contrast, informal governance covers processes and structures related to informal relationships and participants’ social behavior [92]. Therefore, understanding these informal and formal structures and processes is crucial to enable actors to set the collaboration’s overall direction [82]. One of the most famous approaches to illustrate these structures and processes is Tennyson’s parenting life cycle, as illustrated in Fig 1 [93]. This approach is divided into 12 steps with four main phases. The details of each phase are provided below.

#### 2.2.1. Scoping and building

This phase consists of four steps: scoping, identifying, building, and planning. In the scoping step, partners define the area of interest by identifying geographic, thematic, and strategic priorities. Furthermore, partners assess the partnership’s viability through a contextual analysis and map the stakeholders (step #2) [52]. In the building step, the actors jointly define the principles and ground rules for the partnership [94]. At the end of this step, the framework of the partnership is developed (objectives, success indicators of the partnership, deliverables and expected results, roles and responsibilities of each party, the decision-making process, key milestones during the partnership, type of engagement from other stakeholders, and monitoring and evaluation methods) [93]. Once both partners have accepted the partnership proposal, the partnership will be formalized through the signing of legal forms (agreements or memoranda of understanding) [95].

#### 2.2.2. Managing and maintaining

This phase includes three steps: structuring, mobilizing, and delivering. In the structuring step, actors establish the communication protocols, decision-making mechanisms, governance structures, and accountability procedures [93]. The parties allocate financial resources and human resources or mobilize those resources from an external entity. Furthermore, partners should increase the engagement of other stakeholders, including those who may provide further resources (step #6) [94]. The delivering step is considered a continuous cycle of activity [96]. During this step, the partners allocate the roles and responsibilities for program or service delivery as planned, jointly manage the activities, monitor progress for success, and keep partners and other stakeholders informed of their progress [97].

#### 2.2.3. Reviewing and revising

In this phase, partners judge the “health” of the partnering relationship. In other words, they measure the outcomes of the partnership to determine its performance (step #8) [96]. In step #9, partners review the partnership as a basis for confirming the value of the partnership. The purpose of reviewing is to determine whether the partnership is operating efficiently and whether or not it meets their underlying interests [98]. During the revising step (step #10), suggestions for improvements in the partnership are provided. Partners should agree as a group about what needs to be changed, agree on a timetable and change management process, and apply the agreed-upon changes [52].

#### 2.2.4. Sustaining outcomes

In the final phase, depending upon the partnership’s outcomes, partners might decide to end the partnership (either because it has completed its tasks or because it is not delivering sufficient value) or continue it [94]. If the partnership is successful, the partners should consider how to scale it up to facilitate greater reach, impact, and influence [96]. Activities in the scaling step include expanding the established partnerships and publicizing their outcomes using various communication channels. In the final step of the cycle, partners decide whether to move on or not [98]. This step involves a number of options, including ending the partnership with actors free to work with new actors on other projects, continuing to work together as a partnership on new projects, or converting the partnership to a new mechanism or “institution” with its own independent strategy and structure [93].

## 3. Methods

### 3.1. Survey design

A quantitative research methodology using a survey design was adopted as the overarching research strategy for the present study. Quantitative research generally investigates why and how phenomena happen or exist in social life [99]. This study’s primary purpose was to describe the governance of the partnerships by NPOs and examine the differences in governance practices between these organizations. This purpose called for applying a quantitative research design for collecting and analyzing numerical data. By conducting a survey design, researchers can describe patterns in a sample’s opinions to generalize the results or make claims about the population [100]. Furthermore, a survey is appropriate for describing the nature of the current situation of phenomena, identifying the standards against which existing conditions can be compared, or determining the relationships between specific events [101]. The type of survey design adopted for the study was cross-sectional. This survey was conducted in situations where the researcher intended to collect data at one point in time, evaluate various variables at a particular time, and detect the patterns of association [102].

### 3.2. Participant selection

The present study considered one NPO that provides social services in Saudi Arabia and meets all the inclusion and exclusion criteria as a unit of analysis. These criteria included organizations being a registered charity under the Saudi charities regulation of 1990 or the modified regulation of civil organizations of 2015, having an operating age of at least three years as of 31 December 2015, and being engaged in three partnerships during the period of 2016–2018. Even though the research design may also apply to other types of NPOs, they were not considered the unit of analysis in this study. This study identified these criteria to achieve its objectives for the valid and reliable measurement of the phenomena under investigation according to the scale adopted. While the survey was addressed among the organizations’ managers, these managers were not a unit of analysis even though they provided information regarding their organizations’ governance practices, as this study describes an NPO’s governance in a partnership throughout the partnership’s life cycle, not each manager’s characteristics or practices.

### 3.3. Population and sample

This study’s population was the total number of social service NPOs in Saudi Arabia that are registered charities under the charities regulation of 1990 or the civil organization regulation of 2015 and whose details were available in the database of the Ministry of Human Resources and Social Development. In 2020, there were 1335 total social service organizations, including charities [103]. However, the population size was hard to determine because new organizations are constantly being added to the population. Moreover, not all the organizations in the database were able to meet the inclusion criteria. Because of these methodological and practical limitations, this study did not determine the size of the population units but instead drew a sampling frame from the NPO database to make the sample more representative. To determine the accuracy of the sampling frame, we checked the details of registered organizations in the database (name, registration, contact information, and field of practice) and made telephone calls to obtain information about the number of partnerships signed during 2016–2018. The final sampling frame included 498 organizations that met the inclusion criteria. Each organization was listed with serial numbers from 1 to 498. Simple random sampling was employed to select a sample using Yamane’s [104] sample size determination formula. A total of 222 organizations were selected as the intended samples for this study. Accordingly, the questionnaires were sent to these organizations. A total of 184 completed questionnaires were returned to the researchers. This means that an 83% response rate for the survey was achieved.

### 3.4. Data collection instrument

The questionnaire comprised two sections as presented in S1 File. Section one included a profile of the organizations in terms of their field of service, establishment date, region, number of partnerships signed during 2016–2018, and types of actors involved in the partnerships. Section two consisted of governance practices over the life cycle of the partnerships. NPOs were asked to determine each practice’s implementation level during the partnerships’ life cycle on a five-point scale (in all partnerships, in most partnerships, in some partnerships, in a low number of the partnerships, and none). The index of governance practices over the life cycle of the partnerships included 45 items divided into four phases: building and scoping (17 items), managing and maintaining (eight items), reviewing and revising (13 items), and sustaining outcomes (seven items). Each item of the scale represented a widely recommended management practice for the phases in the partnering life cycle framework, and each NPO was asked to rate the extent to which they follow these practices to determine how well these organizations govern their partnerships. To understand the level of implementation in each of these practices, a total scale score was calculated by summing their ratings (all = 5, majority = 4, some = 3, low = 2, none = 1) for each practice. The total score of each step and each phase of the scale was divided into three categories based on the percentage of total scores as follows: low level (<50%), medium level (50%–75%), and high level (>75%).

To ensure the reliability of the instrument, this research adopted internal consistency as its reliability measure by calculating the Cronbach’s alpha coefficient. The value of the Cronbach’s alpha of the scale was 0.82 (>0.7), indicating good internal consistency and high reliability. In terms of content validity, each item included in the scale was measured and operationalized based on the definitions and explanations provided in previous studies that highlighted the partnership life cycle [52, 93–98, 105–108]. Furthermore, each item was examined based on its study purpose and the relevance of the questionnaire content by three managers of NPOs and three academics with related academic backgrounds. Pre-testing of the questionnaire with 10 NPOs in the sampling frame before data collection also assisted in ensuring content validity. According to the responses and suggestions from the managers of the NPOs who participated in the pre-testing, one closed-ended question was removed. Additionally, six items were removed from the scale, as they were not relevant to social service NPOs in Saudi Arabia. All organizations involved in the pre-test were not included in the sampling process. Consequently, the scale employed in this study reached the prescribed standards of reliability and content validity. This study was conducted in accordance with the Human Ethics Committee of King Saud University. Accordingly, this committee, as per the approval memo Ref# KSU-HE-21-38, provided an ethics approval to conduct this study.

Data were collected by face-to-face interviews and by online survey during the period from June to October 2020. An information sheet explaining the purpose of the study and the contact details of the researchers was provided with the questionnaire. The e-questionnaires were shared with the managers of the organizations who were selected through a sampling process via e-mail and WhatsApp. The questionnaires were addressed to the managers of the surveyed NPOs (also known as chief executive officers) who manage the day-to-day affairs and partnership affairs in these organizations, as revealed during the pre-test.

The sample NPOs were given four weeks to fill in the questionnaire, and 43 questionnaires were received back in this period without any reminders. A reminder was then sent to all non-responding NPOs after four weeks. A further two weeks were given to complete the questionnaires after this reminder. Another 54 questionnaires were then received. A final reminder was sent to the sample with another two weeks given to complete the questionnaires, and 29 responses were delivered within this period. This provided a total of 126 responses. The rest of the sample (58 responses) was collected through personal interviews.

### 3.5. Data analysis

The collected data were analyzed using the Statistical Package for Social Sciences (IBM SPSS, ver. 25.0, Armonk, NY: IBM Corp.). Percentages, means, and standard deviations were used as descriptive statistics to describe the data. Inferential statistics of variance analysis (ANOVA) were employed to find the differences between groups. Moreover, the least significant difference test (LSD) was performed to determine which specific means were significant from the others.

## 4. Results

### 4.1. Profile of participant organizations

Table 1 shows the characteristics of the studied NPOs. The participant organizations were divided into six regions of Saudi Arabia. Twenty-nine percent of the NPOs were based in the Makka Al-Mukarramah region, less than quarter (23.9%) were from the Riyadh region, and 15.2% were from the Asir region. Out of the 184 organizations studied, 67 organizations (36.4%) offered in-kind and financial assistance for poor and low-income people. Twenty percent of these organizations provided health services and helped people with disabilities. Furthermore, 15.8% of these organizations identified themselves as providers of family and child welfare services for the population. Half of the participant organizations were registered as civil society organizations under the Saudi civil organizations regulation of 2015. The remaining NPOs were registered as charities under the Saudi charities regulation of 1990. The average years of establishment among these organizations totaled seven years. In terms of the number of partnerships signed during 2016–2018, the findings in Table 1 reveal that the majority of NPOs (71.2%) engaged in fewer than six partnerships, while only 5.4% of these organizations engaged in more than 10 partnerships with various actors. Out of the 937 partnerships identified by the participant organizations, 532 partnerships (56.8%) continued their partnerships for one year. The next most popular timescale for partnerships was less than one year, as observed in 32.1% of the partnerships. Periods of more than one year to two years and more than one year to two years were also observed in 9.4% and 1.7% of the partnerships studied, respectively. Regarding the type of partners engaged with NPOs, the results showed that some organizations engaged with various actors, including other NPOs, charitable foundations, the private sector, the government, and universities. The results indicated that intra-sector partnerships were present in 62.5% of these organizations. Cross-sector partnerships were also observed with the private sector (57.6%), government (54.9%), and universities (3.8%). Finally, tri-sector collaboration was observed in five percent of the total organizations analyzed.

**Table 1:** Profile of participant nonprofit organizations.

Variable	Number of NPOs = 184	
	Frequency	%
Region		
Riyadh	44	23.9
Makka Al-Mukarramah	54	29.4
Al-Madinah Al-Munawwarah	18	9.8
Al-Qassim	17	9.2
The Eastern	23	12.5
Asir	28	15.2
Field of practice		
Poverty alleviation	67	36.4
Health and Disability	37	20.1
Community development	22	11.9
Family and child welfare	29	15.8
Youth	13	7.1
Elderly	12	6.5
Voluntarism	4	2.2
Year of establishment (Min. = 5; Max. = 22; mean = 7.0; SD = 3.16)		
Fewer than 6 years	92	50.0
6–10 years	72	39.1
More than 10 years	20	10.9
Number of partnerships (Min. = 3; Max. = 24; sum = 937; mean = 5.09; SD = 3.05)		
Fewer than 6 partnerships	131	71.2
6–10 partnerships	43	23.4
More than 10 partnerships	10	5.4
Timescale of the partnerships*		
Less than one year	300	32.1
One year	532	56.8
More than one year–two years	89	9.4
More than 2 years	16	1.7
Partners**		
Other NPO(s)	34	18.5
Charitable foundation***	81	44.0
Government	101	54.9
University/ Research institutions	7	3.8
Private sector	106	57.6
Government and private sector	9	4.9

* The percentages calculated according to the number of partnerships;

** More than one answer was allowed; percentages do not add up to 100;

*** organization established by donations of funds that give grants to other organizations.

### 4.2. NPOs’ implementation of partnering life cycle practices

#### 4.2.1. Scoping and building phase

**Fig 2:**
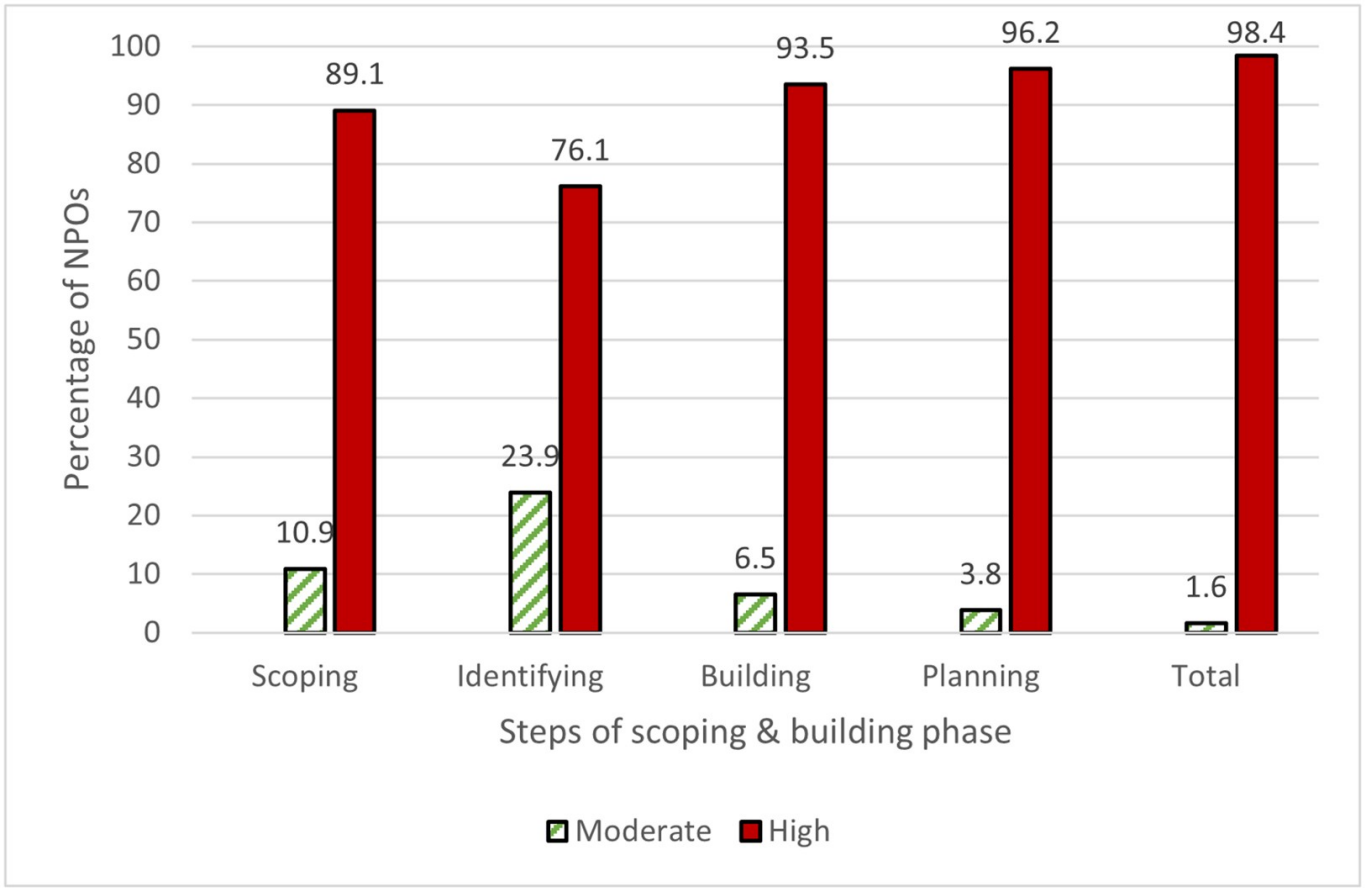
Nonprofit organizations’ implementation level in the scoping and building steps.

The NPOs’ implementation of the partnering life cycle framework in the scoping and building phase is presented in Table 2. Overall, NPOs had a high implementation level, with a percentage of 98.4% (Fig 2). The assessment of the items pertaining to the scoping step (Table 2) shows that organizations considered "identifying the issue(s) to be addressed in the partnership" (mean = 4.80; SD = 0.39), "building a clear rationale to persuade the partners about the importance of collaboration to address the issue specified" (mean = 4.53; SD = 0.70), and "preparing initial ideas about the partnership’s program(s) as a basis for discussion with potential partners" (mean = 4.61; SD = 0.66) as being commonly implemented, whereas they adopted other items less often, which they indicated as being moderately implemented. In general, the findings shown in Fig 2 show that the overwhelming majority of organizations (89.1%) strongly implemented the scoping items examined, while 10.1% of these organizations moderately implemented these items.

**Table 2:** Descriptive results of scoping and building practices followed by nonprofit organizations.

Practices	Mean	Std. Dev.	Rank within each step	Overall rank
Scoping				
Identifying the issue(s) to be addressed in the partnership.	4.80	0.39	1	1
Building a clear rationale to persuade the partners about the importance of collaboration to address the issue specified.	4.53	0.70	3	8
Preparing initial ideas about the partnership’s program(s) as a basis for discussion with potential partners.	4.61	0.66	2	4
Analyzing the different contributions of different actors based on their likely interests and motivations.	3.72	1.05	4	15
Identifying				
Seeking out a wide range of possible partners.	4.20	0.76	2	12
Drawing up a list of preferred partners.	4.58	0.66	1	5
Assessing partners’ suitability in more detail based on a specific set of criteria.	4.15	0.88	3	14
Making initial contact with potential partners on a ’no commitment’ basis to explore the partnership idea.	3.64	1.05	4	16
Building				
Creating opportunities to know more about each of the partners.	4.36	0.69	2	9
Sharing understanding of, and commitment to, the goal among all potential partners.	4.66	0.47	1	2
Exploring how the perceived benefits of the partnership outweigh the perceived costs.	4.32	0.83	3	10
Co-creating some ground rules to support considerate behaviour between the partners.	4.30	0.80	4	11
Planning				
Co-agreeing about the key issue(s) to be addressed by the partnership (stakeholders, focus areas, and specific goals).	4.63	0.48	1	3
Co-agreeing about the outcomes from the partnership’s activities	4.57	0.52	2	6
Co-agreeing about how the achievement of outcomes will be measured and assessed.	3.39	0.96	5	17
Exploring the activities and programs that should be developed to achieve the outcomes	4.56	0.49	3	7
Assessing what resources are needed (human, financial, competencies, etc.) and what each of the partners is able and willing to contribute.	4.19	0.86	4	13

In terms of the identifying step, however, the importance of making initial contact with potential partners to explore the partnership idea had a moderate level of implementation. Table 2 highlights that this practice was the least implemented by organizations in this step (mean = 3.64; SD = 1.05). By contrast, other identifying items were strongly followed. More than three-quarters of organizations (76.1%) strongly implemented the identifying items examined, while 23.9% of these organizations moderately implemented these items (Fig 2).

The results shown in Table 2 indicate that organizations strongly implemented items in the building step. The items with the highest relevance rankings in order of implementation (Table 2) were sharing the understanding of, and commitment to, the goal among all potential partners (mean = 4.66; SD = 0.47); creating opportunities to know more about each of the partners (mean = 4.36; SD = 0.69); exploring how the perceived benefits of the partnership outweigh the perceived costs (mean = 4.32; SD = 0.83); and co-creating ground rules to support considerate behavior between the partners (mean = 4.30; SD = 0.80). To sum up, 93.5% of participant organizations reported a strongly implemented building step, while the remaining noted a moderate level of implementation (Fig 2).

In the planning step, for all five items being assessed (Table 2), the implementation levels ranged from moderate to high. Organizations rated "Co-agreeing about the key issue(s) to be addressed by the partnership" as having the highest level of adoption (mean = 4.63; SD = 0.48), while "Co-agreeing about how the achievement of outcomes would be measured and assessed" had the lowest level of adoption (mean = 3.39; SD = 0.96). Overall, the results shown in Fig 2 show that the majority of NPOs studied (96.2%) had a higher level of implementation of the planning step.

#### 4.2.2. Managing and maintaining phase

**Fig 3:**
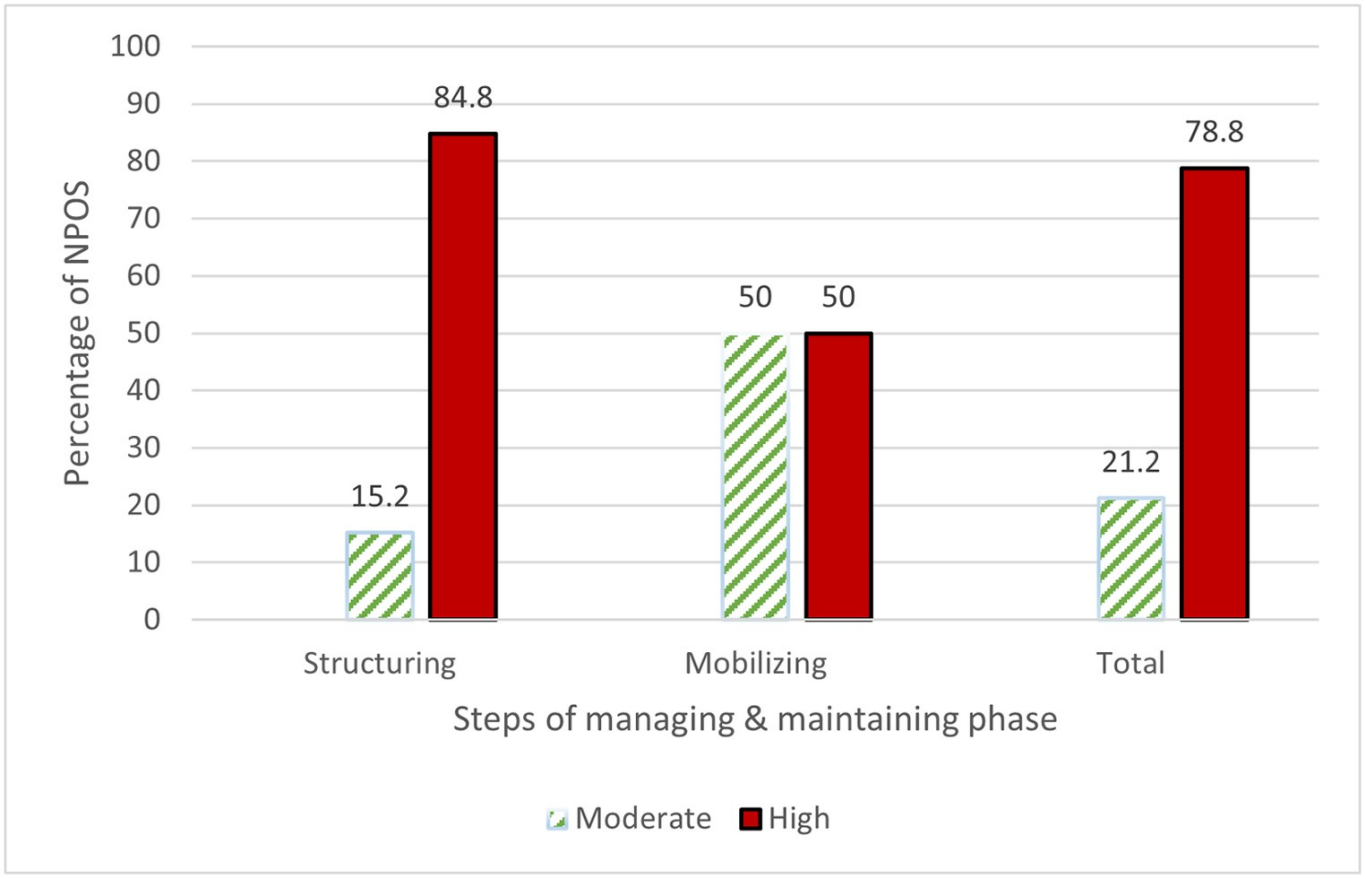
Nonprofit organizations’ implementation level of managing and maintaining steps.

The implementation of managing and maintaining practices as the second phase of the partnering life cycle is highlighted in Table 3. Overall, NPOs had a high implementation level for this phase, with a percentage of 78.8% (Fig 3). All items pertaining to the structuring step (Table 3) were strongly implemented by the organizations (mean ≥ 3.8). For the mobilizing step, the results shown in Table 3 demonstrate that organizations considered widening the engagement of other stakeholders, including those that may be able to provide further resources, as having a medium level of adoption (mean = 2.93; SD = 0.81), while the remaining items were considered to have a high level of implementation by the organizations. Overall, organizations featured highly adopted structuring practices, with 84.4% and 15.2% of these organizations, respectively, strongly and moderately implementing these items (Fig 3). The findings in Fig 3 also reveal that the implementation of mobilizing practices was equally divided between high and moderate levels.

**Table 3:** Descriptive results of managing and maintaining practices followed by NPOs.

Practices	Mean	Std. Dev.	Rank within each step	Overall rank
Structuring				
Identifying and understanding the roles, responsibilities, and expectations of partners.	4.49	0.57	2	2
Establishing the administrative, communication, and decision-making structure of the partnership.	4.59	0.50	1	1
Building an accountability system and addressing any actual or potential conflicts of interest.	3.67	0.77	4	7
Maintaining regular communication between partners and between the partnership and other stakeholders.	4.09	0.64	3	4
Mobilizing				
Determining what resources have been pledged and when they will be delivered, including the time commitments of each partner representative.	4.08	0.67	2	5
Supporting partners in honouring their commitments; helping them persuade their organisations to fulfill their commitments where necessary.	3.97	0.80	3	6
Setting up a system for recording contributions and the implemented applications of those contributions.	4.39	0.71	1	3
Widening the engagement of other stakeholders, including those that may be able to provide further resources.	2.93	0.81	4	8

#### 4.2.3. Reviewing and revising phase

**Fig 4:**
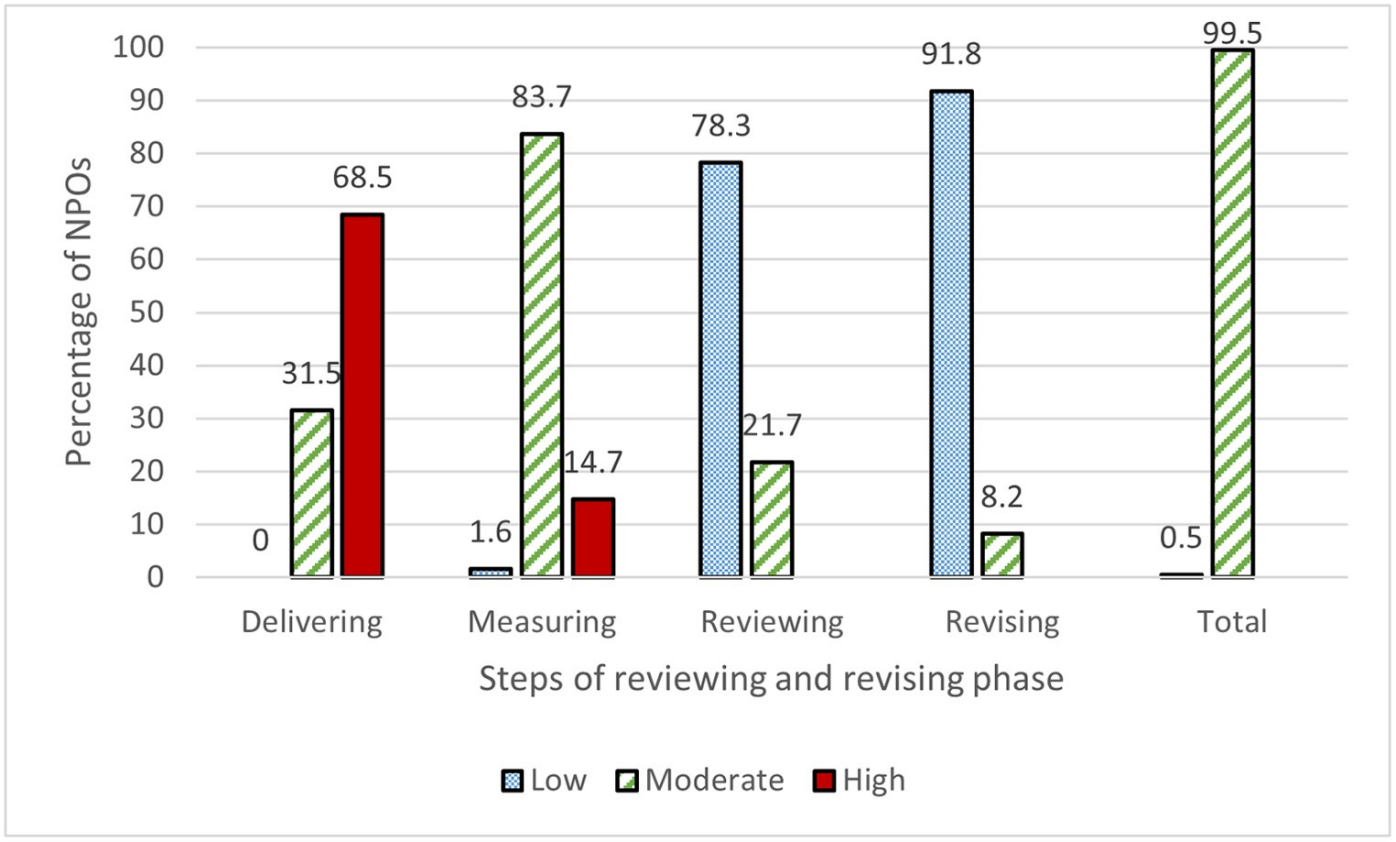
Nonprofit organizations’ implementation level of the reviewing and revising steps.

Table 4 presents the means and standard deviations of the NPOs’ implementation of reviewing and revising practices. As shown in Fig 4, 99.5% of the organizations moderately adopted the steps included in this phase. The details of each step are provided below.

**Table 4:** Descriptive results of reviewing and revising the practices followed by nonprofit organizations.

Practices	Mean	Std. Dev.	Rank within each step	Overall rank
Delivering				
Allocating roles and responsibilities for the program’s delivery.	4.22	0.69	1	1
Providing programs/services for beneficiaries according to their identified needs and priorities.	3.73	0.91	4	5
Tracking activities and fulfillment of agreed-upon commitments and timetables.	3.86	0.77	3	3
Keeping partners and other stakeholders informed of progress	4.27	0.75	2	2
Measuring				
Monitoring the implementation of activities within the agreed-upon timescale.	3.85	0.73	1	4
Keeping track of deliverables and outputs during the partnership.	3.54	0.78	2	6
Determining the partnership’s success by verifying the key performance indicators.	2.7	0.84	3	7
Reviewing				
Determining whether new opportunities/changes could be implemented to ensure the efficiency and effectiveness of the partnership.	2.60	0.77	1	8
Recording any unexpected benefits or outcomes (e.g., wider influence) from the partnership.	2.14	0.67	2	9
Assessing the impact of the partnership among the partners and other stakeholders.	1.83	0.73	3	10
Revising				
Co-agreeing upon what needs to be changed.	1.90	0.69	1	11
Co-agreeing upon a timetable and change management process-allocating tasks between the partners.	1.89	0.79	2	12
Coordinating with partners to implement the agreed-upon changes.	1.77	0.67	3	13

In the delivering step, the assessment of the two statements pertaining to this step (Table 4) demonstrates that organizations considered the implementation of delivering practices (i.e., providing programs/services for beneficiaries according to their needs and priorities identified and tracking the activities and fulfillment of agreed-upon commitments and timetables) as being of a moderate level (2.5 > mean < 3.8). The remaining two items in this step were strongly implemented (mean ≥ 3.8). Overall, more than two-thirds of the organizations (69.5%) strongly implemented the delivering step, while 31.5% of these organizations had a moderate level of implementation.

In the measuring step, all items were considered to have a moderate level of implementation (Table 4). The items with the highest relevance rankings in order of implementation were monitoring the implementation of activities within the agreed-upon timescale (mean = 3.85; SD = 0.73), keeping track of deliverables and outputs during the partnership (mean = 3.54; SD = 0.78), and determining the partnership’s success by verifying the key performance indicators (mean = 2.7; SD = 0.84). As shown in Fig 4, the organizations were classified into three levels according to their level of implementation of the measuring step as follows: high (14.7%), moderate (83.7%), and low (1.6%).

For all three items being assessed (Table 4), the implementation level of the reviewing step ranged from low to moderate. Organizations rated "determining whether new changes could be implemented to ensure the efficiency and effectiveness of the partnership" as having the highest level of implementation (mean = 2.60; SD = 0.77), while "assessing the impact of the partnership on the partners and other stakeholders" had the lowest level of adoption (mean = 1.83; SD = 0.73). Overall, the results in Fig 4 indicated that 78.3% of the NPOs studied were classified in the "low implementation" category for the reviewing step, while the remaining organizations were in the "moderate implementation" category.

A low level of implementation of the revising step among NPOs was observed in all items (Table 4). The average mean ranged between a maximum of 1.9 for the item of "co-agreed upon what needs to be changed" and a minimum of 1.77 for "coordinating with partners to implement the agreed-upon changes." Overall, the vast majority of organizations (91.8%) were in the "low implementation" category for the revising step, while 8.2% were in the "moderate implementation" category (Fig 4).

#### 4.2.4. Sustaining outcome phase

**Fig 5:**
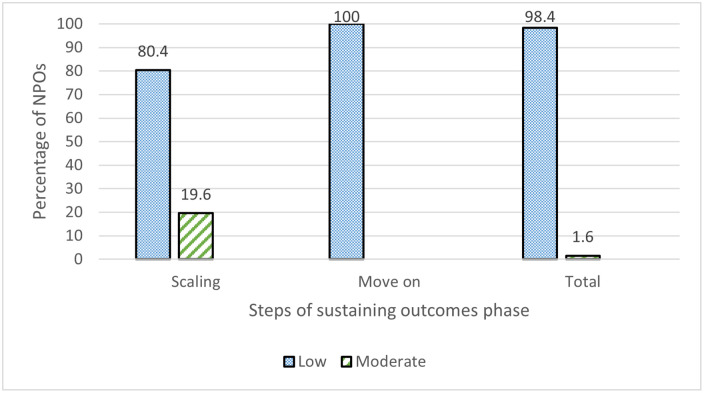
Nonprofit organizations’ implementation levels for the sustaining outcome steps.

All seven statements assessed for scaling and moving the steps involved in this phase were considered to have low implementation for sustaining outcomes (Table 5). As shown in Fig 5, the overall mean of the vast majority of organizations (80.4%) was low regarding the implementation of the scaling step, while the remaining organizations had a moderate level of implementation. In terms of the moving-on step, all organizations had a low level of implementation.

**Table 5:** Descriptive results of sustaining outcome practices followed by nonprofit organizations.

Practices	Mean	Std. Dev.	Rank within each step	Overall rank
Scaling				
Partners agreed on expanding the established programs.	1.80	0.76	3	3
Using media and social media for publicizing the results and impacts.	2.29	1.10	1	1
Summarizing the partnership lessons learned and making them available to other NPOs.	2.00	0.85	2	2
Encouraging other NPOs to adopt a partnering approach.	1.53	0.66	4	4
Moving on				
Continuing to work together as a partnership on new programs.	1.63	0.55	1	5
Continuing to work alone or with new partners based on resources available from either internal or external sources.	1.2	0.45	2	6
Co-developing core-business enterprises with their own independent strategies and structures based on the success of the partnership.	1.05	0.32	3	7

**Fig 6:**
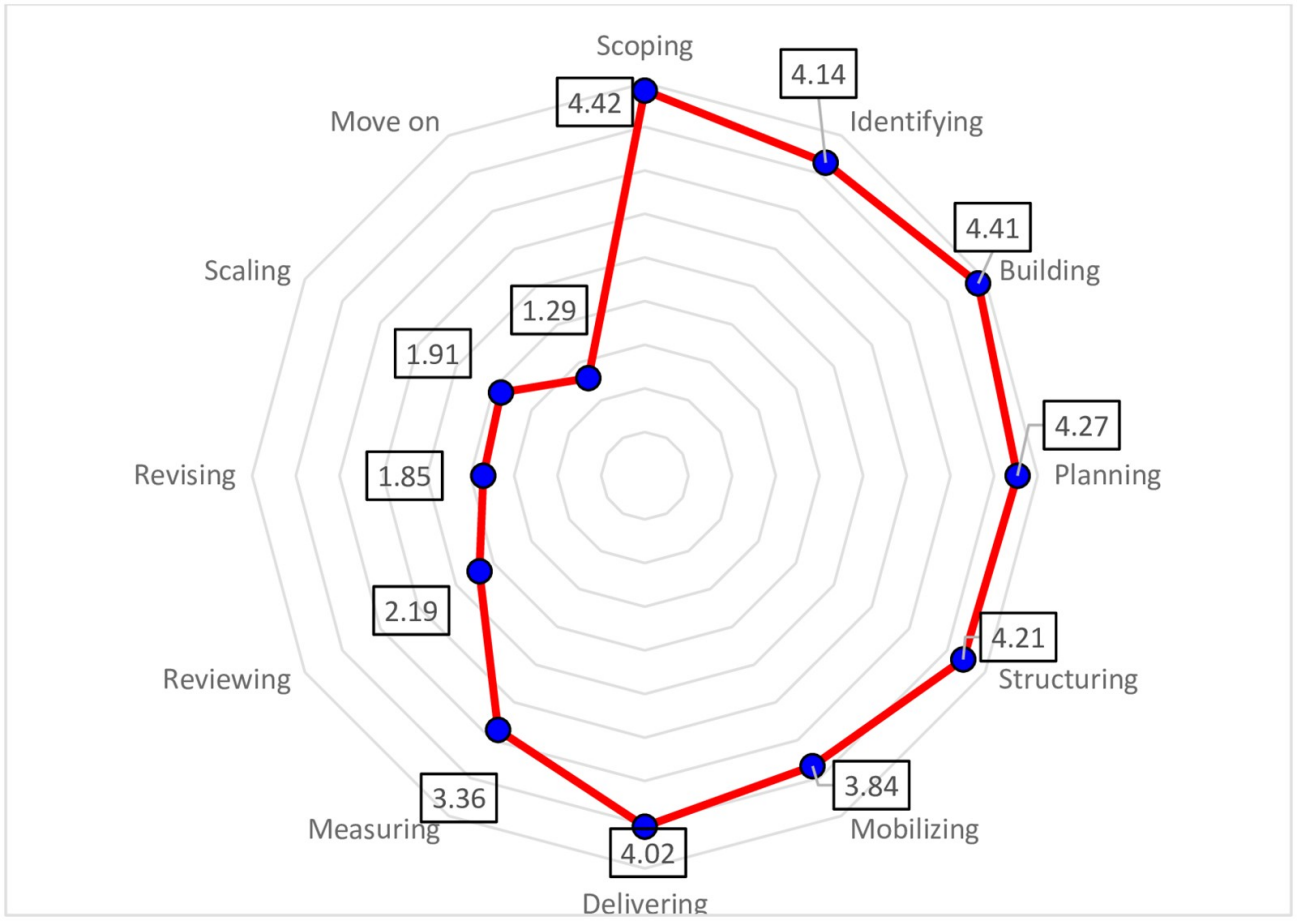
Summary of nonprofit organizations’ implementation of partnering life cycle practices.

The overall mean of each step of the partnering life cycle framework is presented in Fig 6. Out of the 12 steps investigated, seven steps were strongly implemented by organizations, one step had a moderate level of implementation, and four steps had a low level of implementation among the organizations. In general, organizations had good expertise in governing the partnerships during the phases of building and scoping and managing and maintaining. On the other hand, these organizations still need to enhance their governance practices for the phases of reviewing and revising and sustaining outcomes to create sustainability-oriented partnerships.

Table 6 shows the general implementation patterns of the partnering life cycles among the participant NPOs based on the overall mean responses obtained during the survey. The results indicate that the majority (99.5%) of organizations were in the "moderate implementation" category, whereas only one organization (0.5%) was in the "high implementation" category.

**Table 6:** Distribution of nonprofit organizations’ according to their implementation level of partnering life cycle practices.

Implementation Level	Number of Organizations = 184	
	Frequency	Percentage (%)
Low (fewer than 50%)	-	-
Moderate (50%–75%)	183	99.5
High (more than 75%)	1	0.5

### 4.3. Differences in the implementation of partnering life cycle practices between NPOs

Table 7 shows the differences in the overall implementation of governance practices over the life cycle of the collaboration between partners based on the characteristics of NPOs. The F test results reveal that significant differences existed between the overall implementation of partnering life cycle practices and the NPO’s year of establishment at *p* < 0.05. This result demonstrates that the NPOs differed in their implementation of governance practices based on their establishment date. According to the results of the LSD test, the mean value of the "6–10 years" category significantly differed from that of the "fewer than six years" category at the 0.05 level. Moreover, the mean value of the "more than 10 years" category significantly differed from the "fewer than six years" category at the 0.01 level. This indicates that the organizations with a longer period of establishment scored higher in the items related to implementing governance practices included in the partnering life cycle. The findings also showed no significant differences in the overall implementation of governance practices regarding the region, field of practice, or number of partnerships.

**Table 7:** Differences in overall implementation of the partnering life cycle practices based on nonprofit organizations’ characteristics.

Variable	F	Sig.	Least significant difference test			
			Mean Difference		Std. Error	Sig.
Region	0.53	0.75				
Field of practice	1.13	0.34				
Year of establishment	3.96	0.02*	<6 years–6–10 years	-0.74	0.38	0.05*
			6–10 years–> 10 years	-0.73	0.61	0.23
			<6 years–>10 years	-1.48	0.59	0.01**
Number of partnerships	0.05	0.94				

** *p* < 0.01;

* *p* < 0.05; Sig. = level of significance.

## 5. Discussion

In this article, we examined the formal and informal processes of partnerships through which NPOs and other actors frame and prioritize issues, articulate their interests, and make, implement, monitor, and enforce decisions. The insights from this exploration will enrich existing literature on the governance of partnerships, which is mainly based on qualitative research. From a theoretical point of view, partnerships with strong internal governance are more likely to leave a social footprint in society and encourage partners to engage in sustainable collaboration with NPOs. This study assessed how partnerships between NPOs and other actors in Saudi Arabia should be practically managed for sustainability by adopting the partnering life cycle framework. This approach provides a series of valuable guidelines to analyze the phases and steps necessary to form a partnership. Furthermore, it gives a complementary vision to design healthy relationships between partners to increase the transformation potential of a partnership. Our results about the current situation of applying the steps included in the partnering life cycle framework complement existing knowledge and offer insights into how we can increase the efficiency and impact of working in partnerships.

Our findings highlight the increasing linkages between NPOs and the development of cooperating actors. The number of partnerships signed during 2016–2018 was promising and indicates a substantial transformation of collaboration to meet mutual interests and solve complex issues. This transformation reflects the Saudi government’s significant role through Vision 2030 in supporting partnerships between NPOs and other actors [62]. After announcing this vision in 2016, many governmental initiatives, in terms of contractual agreements, were provided for actors to engage in partnerships [58]. The aim of these initiatives was to help NPOs become more business-like [17]. Maier, Meyer [23] summarized the effects of NPOs becoming business-like in four areas: improving their organizational performance; fulfilling their societal functions; gaining power and knowledge; and increasing their legitimacy. On the other hand, as indicated by Kuruvila [11], management approaches developed in the private sector are not appropriate for NPOs due to their mission-driven nature and value orientation. However, the limited availability of funding results in NPOs not being able to provide holistic services to their stakeholders. This environment is also informed by the new public management approach, which promotes outcome perspectives and audit culture in service delivery, forcing NPOs to adopt a business management approach to enhance their performance [109]. As a result of these transformations, actors seek to exploit synergies in two dimensions: achieving multiple SDGs that generate significant co-benefits [110] and strengthening governance structures [111].

Prior to signing a partnering agreement, the results showed that NPOs professionally undertake the needed steps to form a partnership. These steps relate to four areas: identifying the issue to be addressed, identifying appropriate partners, suggesting ground rules for building successful partner relationships, and outlining a planning and resource mapping framework. These results confirm the availability of an environment conducive to partnering. In this context, Sterne, Heaney [112] indicated that creating suitable conditions for sustainability-oriented partnerships is dependent on the external and internal environment. The external environment includes sociopolitical factors that promote the formation of a partnership, including a regulatory decline in governmental efficacy, meeting the expectations of the stakeholders, increasing the role of information communication technology in sustainable development, and the absence or ineffectiveness of regulatory frameworks [32, 37, 48, 69]. Internal factors also collectively motivated the actors to address an issue or set of issues [112]. As argued by Gray and Stites [46], four main motivations are gained from partnerships between NPOS and other actors, including leveraging resources, competencies, legitimacy, and responding to stakeholder problems. The existence of such factors encourages actors to establish new institutional arrangements to improve their capacity to deal with sustainability issues [113].

Some difficulties were observed during the implementation of the partnerships. Meeting stakeholders’ needs and mapping stakeholders’ priorities are two of the challenges that NPOs face in the delivering step. Furthermore, the findings revealed that NPOs’ implementation of the reviewing and revising steps ranged from low to moderate levels. These results show that some governance practices still need to be enhanced to minimize their impacts on a partnership’s effectiveness and achieve social innovation. Practically, some NPOs could face such problems due to a lack of required competencies, the nature of the issue to be addressed, and the level of engagement with partners in performing activities [6]. In this regard, previous studies emphasized the role of third parties as brokers that play a crucial bridging role in balancing partners’ interests [9, 84, 114, 115]. As indicated by Stadtler and Probst [115], brokers can adopt three roles: a convener, a mediator, or a learning catalyst. More specifically, brokers can play these roles during the life cycle to help partners overcome challenges that jeopardize the successful partnering process [115]. Therefore, to enable third parties to make the partnership process successful, the broker should be aware from the beginning of the collaboration in its role and how willing it is to achieve this potential [9].

Sustaining outcomes was the least commonly implemented phase by NPOs. As evidenced from the results, the activities included in the steps of scaling and moving on were rarely implemented by NPOs throughout the life cycle of partnerships. This result may be because most of the partnerships signed were characterized as philanthropic, rather than strategic. This would reduce the willingness of partners to develop long-term mechanisms for sustaining outcomes with NPOs. Furthermore, most partnerships, as shown in Table 1, continued for one year or less. This limited period of partnerships was not a positive prerequisite for sustaining value creation. These results are in line with the results of Kassem, Aljuaid [62], who showed in their map of 459 partnerships between NPOs and other actors in Saudi Arabia that 89.7% of the partnerships could be called "transactional partnerships" in cases of both philanthropic and social investment partnerships and that only 8% of these partnerships continued after their end date or were converted to core-business enterprises so as to provide long-term benefits for partners. Globally, NPOs are operating in rapidly changing, ambiguous, complex, paradoxical, and multicultural contexts [116]. NPOs are currently operating in an environment affected by a neoliberal approach that encourages competition among them to deliver social services through partnering with the private sector and governments; this process does not ensure sustainability and continuity for the clients who receive the related services [109]. This quasi-market delivering system is putting massive pressure on NPOs to demonstrate their accountability and efficient use of limited resources to the government while the demand for services is on the rise [117].

The extent to which the NPOs followed governance practices varied among these organizations in Saudi Arabia based on their year of establishment. Kuruvila [11] argued that less-experienced NPOs struggle with their functioning and internal management of partnerships and need to build their management capacities to navigate their relationships with partners. These challenges in Saudi Arabia were related to a shortage of professionals qualified to handle cross-sector partnerships; a lack of expertise in establishing governance principles; a lack of co-ordination and networking skills with various actors; and the weakness of transparency and accountability measures [61, 118]. In this context, Huyse, Phlix [119] concluded that less-experienced NPOs should invest in a clear vision of the nature of the capacities that they wish to build. Such capacities will further develop the quality of their partnerships and facilitate sustainable outcomes.

This study had some limitations that should be acknowledged. First, this study focused only on social service organizations registered as charities under the charities’ regulation of 1990 or the civic organizations regulation of 2015. Although our results provide some insights into the partnership governance of NPOs in general and may assist other types of NPOs, these results are mainly limited to social service organizations. While the study results may be useful to NPOs in other countries, this research’s generalization is limited to Saudi Arabia’s NPOs. Second, the data collection tool included close-ended questions to identify the implementation level of governance practices. The responses may have been influenced by social desirability biases, and the method of data collection could have prevented the researchers from obtaining additional comments. Third, data were collected from NPOs as partners in collaborations without obtaining the opinions of other actors that engage with these organizations in the partnerships. This approach is not sufficient to accurately describe the nature of the partnerships’ governance in formal and informal structures or in the processes implemented during collaboration. Fourth, the level of analysis involved only organizations. Highlighting governance practices according to each partnership could give a fuller picture of how these practices differ according to partnerships’ configuration and the types of actors involved in the partnership. Finally, the profiles of the NPOs included only some general characteristics of these organizations and the partnerships that they were involved in with other actors. Other indicators of organizational capacity, such as the board, service delivery, staff, volunteers, and finances, are also important to identify the management of NPOs. Therefore, such variables should be included in future research to explain and interpret the differences—if they exist—among organizations in partnership governance practices.

## 6. Conclusions

This study attempted to develop an understanding of NPOs’ governance of partnerships in Saudi Arabia. This aim was achieved by examining the NPOs’ implementation of the partnering life cycle framework. The story of the NPOs’ implementation of the phases and steps included in this framework could be described as an "hourglass", with promising beginnings and incomplete or non-hopeful endings. As evident in the partnering life cycle analysis, partnership governance is a complex issue that requires the management of internal organizational processes and the organization’s interactions with the external environment. The findings concluded that NPOs still need to enhance their monitoring and evaluation practices to improve their partnerships’ value for society and the partners. Additionally, how NPOs can scale their successful partnerships up and develop long-term mechanisms to sustain their partnerships remains questionable. The practices of governance over the life cycle of the partnership developed in this study have implications in both theory and practice. This paper offers a relatively simple view of NPO governance without losing its complexity. This framework conceptualizes the interactions between partners in both internal and external environments in easily understandable steps and offers a practical guide with a tested and reliable rating scale to assist future researchers who want to research the governance of partnerships. Practically, this study provides insights into the gaps that need to be filled by NPOs during the life cycle of a partnership, particularly during the steps of measuring, reviewing, revising, scaling, and moving on. One of the needs expressed by NPOs is the concern to reach an agreement on how the achievement of outcomes will be measured and assessed and what needs to be changed, hence the importance of dialogue along the life-cycle of the partnership process. Co-developing performance indicators are essential to evaluate not only of the collaboration between parties itself, but also of the partnership’s outcomes. Therefore, the assessment of results should consider three main areas: the partnership’s value for each of the partners involved, the true costs and benefits of the partnership approach, and impacts on the target group in particular and society in general. External third parties are urgently needed as brokers between partners to enhance collaboration practices. Brokers can act as allies to complement the partners’ roles during the entire life cycle of a partnership to enable NPOs to achieve more significant social innovation. Also, the study recommends using social media as a part of organizational strategy of the partnership. The main purpose of such platforms is to enhance NPO’s visibility, publicize the results, and connect with other NPOs for sharing of lessons learned of the partnership. From a policy perspective, as most of the participant NPOs rely on government and private sector contracts for their functioning, new collaborations should consider the capacity-building needs of NPOs in terms of governance practices along a partnership’s life cycle. Due to the scarcity of research on partnership governance in Saudi Arabia’s nonprofit sector, more research in this area is needed to bring more diverse perspectives to the discussion of partnership governance. This study highlights the need for more comprehensive empirical research on the impact of external environmental barriers on partnership governance. Moreover, how the governance of these partnerships may be influenced by the type and number of partners and the organizational capacity of NPOs would be an interesting topic to investigate.

## Supporting information

S1 Data (XLSX)

S1 File (PDF)
