# Supplementary material for: Are partnerships in nonprofit organizations being governed for sustainability? A partnering life cycle assessment
Source: PLoS One. 2021 Mar 29;16(3):e0249228. doi: 10.1371/journal.pone.0249228 (PMC8007011; doi:10.1371/journal.pone.0249228)
Supplement: S1 File — (PDF) [file pone.0249228.s002.pdf]

Questionnaire

Dear respondent, we are conducting research on the governance of partnerships in nonprofit organizations. To complete this research work, I need your voluntary participation in this survey. If you are willing to participate in this study, then I request you to respond to the statements given in this survey. Your valuable response will help us in the completion of this project.

Your name should not appear anywhere in this survey, and your response will be treated confidentially and will only be used for research purposes.

First: General data

1- Region

Riyadh ( )	Makka Al-Mukarramah ( )	Al-Madinah Al-Munawwarah ( )
Al-Qassim ( )	The Eastern ( )	Asir ( )

2- Field of practice

Poverty alleviation ( )	Health and Disability ( )	Community development ( )
Elderly ( )	Youth ( )	Family and child welfare ( )
Voluntarism ( )	Other (Please specify)	

3- Year of establishment ( )

4- Number of partnerships signed during 2016 to 2018 ( )

5- Timescale of the partnerships

Timescale	Number of partnerships
Less than one year	
One year	
More than one year–two years	
More than 2 years	

6- Partners (more than one answer can be applied)

Other NPO(s) ( )	Charitable foundation ( )	Government ( )
University/ Research institutions ( )	Private sector ( )	Other ( )

Second: Governance of partnerships

In this section, please select the response that indicates the degree to which you implemented each statement in all partnerships or not at all (Only one).

NO.	Statements	All	Majority	Some	Low	None
**A. Scoping & Building**						
A1	**Scoping**					
1	Identifying the issue(s) to be addressed in the partnership.					
2	Building a clear rationale to persuade the partners about the importance of collaboration to address the issue specified.					
3	Preparing initial ideas about the partnership's program(s) as a basis for discussion with potential partners.					
4	Analyzing the different contributions of different actors based on their likely interests and motivations.					
A2	**Identifying**					
5	Seeking out a wide range of possible partners.					
6	Drawing up a list of preferred partners.					
7	Assessing partners' suitability in more detail based on a specific set of criteria.					
8	Making initial contact with potential partners on a 'no commitment' basis to explore the partnership idea.					
A3	**Building**					
9	Creating opportunities to know more about each of the partners.					
10	Sharing understanding of, and commitment to, the goal among all potential partners.					
11	Exploring how the perceived benefits of the partnership outweigh the perceived costs.					
12	Co-creating some ground rules to support considerate behaviour between the partners.					
A4	**Planning**					
13	Co-agreeing about the key issue(s) to be addressed by the partnership (stakeholders, focus areas, and specific goals).					
14	Co-agreeing about the outcomes from the partnership's activities					
15	Co-agreeing about how the achievement of outcomes will be measured and assessed.					
16	Exploring the activities and programs that should be developed to achieve the outcomes					

NO.	Statements	All	Majority	Some	Low	None
17	Assessing what resources are needed (human, financial, competencies, etc.) and what each of the partners is able and willing to contribute.					
B	**Managing & Maintaining**					
B1	**Structuring**					
18	Identifying and understanding the roles, responsibilities, and expectations of partners.					
19	Establishing the administrative, communication, and decision-making structure of the partnership.					
20	Building an accountability system and addressing any actual or potential conflicts of interest.					
21	Maintaining regular communication between partners and between the partnership and other stakeholders.					
B2	**Mobilizing**					
22	Determining what resources have been pledged and when they will be delivered, including the time commitments of each partner representative.					
23	Supporting partners in honouring their commitments; helping them persuade their organisations to fulfil their commitments where necessary.					
24	Setting up a system for recording contributions and the implemented applications of those contributions.					
25	Widening the engagement of other stakeholders, including those that may be able to provide further resources.					
C	**Reviewing and Revising**					
C1	**Delivering**					
26	Allocating roles and responsibilities for the program's delivery.					
27	Providing programs/services for beneficiaries according to their identified needs and priorities.					
28	Tracking activities and fulfillment of agreed-upon commitments and timetables.					
29	Keeping partners and other stakeholders informed of progress					
C2	**Measuring**					
30	Monitoring the implementation of activities within the agreed-upon timescale.					

NO.	Statements	All	Majority	Some	Low	None
31	Keeping track of deliverables and outputs during the partnership.					
32	Determining the partnership's success by verifying the key performance indicators.					
C3	**Reviewing**					
33	Determining whether new opportunities/ changes could be implemented to ensure the efficiency and effectiveness of the partnership.					
34	Recording any unexpected benefits or outcomes (e.g., wider influence) from the partnership.					
35	Assessing the impact of the partnership among the partners and other stakeholders.					
C4	**Revising**					
36	Co-agreeing upon what needs to be changed.					
37	Co-agreeing upon a timetable and change management process-allocating tasks between the partners.					
38	Coordinating with partners to implement the agreed-upon changes.					
D	**Sustaining Outcomes**					
D1	**Scaling**					
39	Partners agreed on expanding the established programs.					
40	Using media and social media for publicizing the results and impacts.					
41	Summarizing the partnership lessons learned and making them available to other NPOs.					
42	Encouraging other NPOs to adopt a partnering approach.					
D2	**Move on**					
43	Continuing to work together as a partnership on new programs.					
44	Continuing to work alone or with new partners based on resources available from either internal or external sources.					
45	Co-developing core-business enterprises with their own independent strategies and structures based on the success of the partnership.					

Thanks for your time.
